# Secondary primary tumors following iodine-125 low-dose-rate brachytherapy for localized prostate cancer

**DOI:** 10.1007/s10147-026-03020-3

**Published:** 2026-03-29

**Authors:** Kojiro Niwa, Koji Iinuma, Masahiro Nakano, Masayuki Tomioka, Masaya Ito, Takayuki Mori, Kota Kawase, Tomoki Taniguchi, Yuki Tobisawa, Keita Nakane, Masayuki Matsuo, Takuya Koie

**Affiliations:** 1 Department of Urology, Graduate School of Medicine, Gifu University, 1-1 Yanagido, Gifu, Gifu 501-1194 Japan; 2 Department of Urology, Gifu Prefectural General Medical Center, 4-6-1 Noisiki, Gifu, Gifu 500-8717 Japan; 3 Department of Radiology, Graduate School of Medicine, Gifu University, 1-1 Yanagido, Gifu, Gifu 501-1194 Japan

**Keywords:** Prostate cancer, Low-dose-rate brachytherapy, Secondary primary tumor, Biologically effective dose, Late toxicity

## Abstract

**Background:**

Low-dose-rate brachytherapy with iodine-125 (LDR-BT) is an established curative radiation treatment modality for localized prostate cancer (PCa). This study aimed to evaluate the long-term incidence of secondary primary tumors (SPTs) following LDR-BT in Japanese patients with localized PCa and identify predictive factors associated with SPTs.

**Methods and materials:**

We retrospectively reviewed the clinical records of 478 consecutive patients with localized PCa who underwent LDR-BT at the Gifu University Hospital. This study’s primary endpoint was SPTs incidence, including bladder and rectal/anal cancers. The secondary endpoint was the identification of risk factors for LDR-BT that predicted SPTs development.

**Results:**

After a median follow-up period of 105 months, SPTs developed in 13 patients (2.7%). Bladder cancer and rectal/anal canal cancer were observed in seven (1.5%) and six (1.3%) patients, respectively. Multivariate analysis showed that a biologically effective dose (BED) ≥ 197 Gy was associated with increased risk of SPTs (hazard ratio 4.145; 95% confidence interval 1.108–15.498; *p* = 0.035).

**Conclusions:**

The incidence of SPTs following LDR-BT was relatively low. In multivariate analysis, BED ≥ 197 Gy may be associated with an increased risk of SPT occurrence. Adequate tumor control can be achieved through appropriate radiation dose administration; carefully planned long-term follow-up may be beneficial for SPTs early detection.

## Introduction

Prostate cancer (PCa) is one of the most common malignancies among men worldwide and in Japan [1, 2]. Definitive treatment modalities for localized PCa, including active surveillance, surgical intervention, and radiation therapy (RT), have demonstrated favorable oncological outcomes worldwide [2].

The low-dose-rate brachytherapy with iodine-125 (LDR-BT), which is used in combination with or without external beam radiation therapy (EBRT), is one of the most effective treatment modalities for RT [3]. LDR-BT generally yields favorable oncological outcomes [4–6]. Previous studies from our institution demonstrated excellent long-term oncological outcomes after LDR-BT [4–6]. A median follow-up period of 90.0 months revealed a 10 year biochemical recurrence (BCR)-free rate of 95.3% [6]. Notably, the 10 year BCR-free rate maintained a high level of 93.4% among patients with PCa categorized as high-risk according to the D’Amico classification [6, 7]. Although LDR-BT is an effective treatment for long-term cancer control, there are still significant treatment-related toxicities, including radiation-related late complications involving the genitourinary and gastrointestinal organs, including secondary primary tumors (SPTs) following LDR-BT. SPTs are generally defined as occurring > five years after receiving RT [10–12]. This critical complication should be considered when selecting the most effective treatment for localized PCa. Conversely, the occurrence of late complications, including SPTs, in patients with PCa who underwent RT, particularly LDR-BT, followed by long-term observation, remains limited.

Therefore, this study aimed to evaluate the long-term incidence of SPTs following LDR-BT for localized PCa in Japanese patients. Additionally, an analysis was conducted to determine predictive factors associated with SPTs occurrence.

## Materials and methods

### Patients

We retrospectively reviewed the clinical records of 478 consecutive patients with PCa who underwent LDR-BT at the Gifu University Hospital between August 2004 and December 2019. Patients with localized PCa, without lymph node or distant metastases, were enrolled according to the 2016 American Joint Committee on Cancer Staging Manual [13]. Patients were divided into risk categories according to the National Comprehensive Cancer Network (NCCN) classification [14]. The following clinical data were collected from the enrolled patients: age, body mass index (BMI), initial serum prostate-specific antigen (PSA) level, clinical T stage, biopsy Gleason grade [15], NCCN risk classification, prostate volume (PV) at LDR-BT, presence or absence of androgen deprivation therapy (ADT), and follow-up duration. Colonoscopies were performed before LDR-BT in all patients who had not undergone this examination within the previous two years, in accordance with the protocol initiated in April 2010 [16]. In patients with microscopic hematuria prior to LDR-BT, urinary cytology and cystoscopy were performed to exclude bladder cancer or bladder invasion of prostate cancer. Patients with lymph node involvement, distant metastasis, a history of transurethral resection of the prostate, or a maximum urinary flow rate < 10 mL/s on uroflowmetry were excluded from the study.

Due to the retrospective nature of this study, informed consent was waived, according to the Japanese Ethical Guidelines. An opt-out approach was implemented, thereby giving patients the opportunity to decline participation. This study was reviewed and approved by the Institutional Review Board of Gifu University (approval number: 29–106).

Although smoking history was not systematically documented for the entire cohort, detailed smoking status was meticulously reviewed for all patients who developed bladder cancer, based on medical records.

### Treatment of LDR-BT

As previously documented, this report details the treatment procedures for LDR-BT at our institution [8, 9, 17]. Patients with low-risk PCa and a PV > 50 mL received neoadjuvant ADT for at least 3 months before LDR-BT. Patients diagnosed with intermediate-risk PCa were administered ADT for 9 months, followed by LDR-BT with or without EBRT. Patients with high- or very high-risk PCa underwent LDR-BT combined with EBRT and ADT for 24 months. During the administration of EBRT, the radiation field was confined to the prostate and seminal vesicles.

Patients were implanted with loose 125I radioactive seeds (Oncoseed, Nippon Medi-Physics, Tokyo, Japan) by the Mick Applicator (Mick Radio-Nuclear Instruments, Bronx, NY, USA) or the ProLink® (Cincinnati, OH, USA) delivery system (C. R. Bard, Inc., Murray Hill, NJ, USA) under real-time confirmation by transrectal ultrasound transperineally into the prostate [18]. A prescribed minimum peripheral dose of 145 Gy was used for LDR-BT monotherapy, and 104 Gy was used when combined with EBRT. When EBRT was used, a total dose of 40 Gy in 2-Gy fractions was delivered to the prostate and seminal vesicles within 1 month after LDR-BT. In all cases, a modified peripheral loading technique was applied after preplanning the seed implantation [19].

Therapeutic planning and post-implant dosimetric evaluations were performed using the updated American Association of Physicists in Medicine Task Group 43 for-malism and Variseed version 7.1 (Varian Medical Systems, Palo Alto, CA, USA). Subsequent dosimetric evaluations were conducted using both computed tomography (CT) and magnetic resonance imaging (MRI) modalities, with assessments performed one month after LDR-BT [20]. The dosimetric parameters that were the focus of this study included the minimum dose received by 90% of the prostate gland (D90), biologically effective dose (BED), minimum percentage of the dose received by 30% of the urethra (UD30), and the rectal volume receiving 100% of the prescribed dose (RV100). UD30 is a postoperative dosimetric parameter that is frequently utilized to evaluate urethral dose exposure in LDR-BT. Previous studies have employed UD30 to assess delayed urogenital toxicity [18]. The BED was calculated using an alpha/beta ratio of 2.

All patients were monitored with follow-up examinations at 3–6 months intervals for 5 years, followed by biennial checkups. The follow-up process included interval history, physical examination, and PSA measurement. Furthermore, MRI scans were obtained 3, 5, and 10 years after LDR-BT. Patients presenting with gross or microscopic hematuria underwent a series of diagnostic procedures, including urinalysis, urinary cytology, and cystoscopy. Patients presenting with bloody stool or other lower gastrointestinal symptoms underwent colonoscopy. These examinations resulted in a diagnosis of SPTs. The follow-up period was defined as the time interval between the completion of RT and the most recent follow-up visit or date of death. BCR definition was established in accordance with the Radiation Therapy Oncology Group-Phoenix classification, which sets the threshold for the PSA nadir at 2 ng/mL [21].

### SPTs following LDR-BT

SPTs were defined as in-field second primary cancers that developed at least 5 years after LDR-BT, including bladder, urethral, rectal, and anal canal cancers [11, 12, 22]. The definition of SPTs utilized in this study was employed to identify secondary cancers that developed within the radiation field after a sufficient latency period. However, it is important to note that this does not imply that all malignant tumors occurring within the radiation field are attributable to radiation exposure, nor can they be clearly distinguished from age-related cancers. SPTs were defined exclusively in cases in which the diagnosis was subsequently confirmed by biopsy or surgical pathology.

### Statistical analysis

The primary endpoint of the study was SPTs incidence, including bladder and rectal/anal cancers. The secondary endpoint was the identification of risk factors for LDR-BT that predicted the SPTs development. Additionally, the incidences of bladder and rectal/anal canal cancers were analyzed. The chi-square test was used to evaluate differences between categorical variables, and the Mann–Whitney U test was used for continuous variables. The incidence rates of SPTs, bladder cancer, and anal canal cancer were evaluated using cumulative incidence proportions, and differences according to clinical variables were assessed using the chi-square test. The Kaplan–Meier method was used to estimate overall survival (OS) and BCR. Cox proportional hazard models were used to assess the relationship between potential predictors of SPTs. To assess the robustness of the association between BED and SPTs, given the limited number of SPTs cases in the primary Cox proportional hazards model, a sensitivity analysis was conducted using a reduced model containing only age and BED. Death was treated as a competing event, and the cumulative incidence function was estimated. The optimal cut-off values for age, BMI, and BED were determined using receiver operating characteristic curve analysis [23]. The data were analyzed using software JMP 14 (SAS Institute Inc., Cary, NC, USA). All statistical analyses were two-sided, and *p* < 0.05 was considered statistically significant.

## Results

### Patient characteristics

Baseline patient characteristics are summarized in Table 1. The median age, BMI, and PSA level of the entire cohort were 66.0 years (interquartile range [IQR]: 62.0–71.0), 23.5 kg/m^2^ (IQR: 21.9–25.3), and 6.5 ng/mL (IQR: 5.1–9.1), respectively. According to the NCCN risk classification, 154 patients were classified as low risk, 126 as favorable intermediate risk, 137 as unfavorable intermediate risk, 54 as high risk, and seven as very high risk. The median BED, UD30, and RV100 were determined to be 194.8 Gy (IQR: 179.1–208.8), 191.2 Gy (IQR: 159.5–218.3), and 0.3 cc (IQR: 0.1–0.7), respectively. The median follow-up period was 105.0 months (IQR: 71.0–134.0). The median overall survival (OS) was found to be 242.0 months. The median BCR-free survival was not reached; however, the 10 year BCR-free survival rate was 94.9%. Notably, only one patient died of PCa.

**Table 1:** Patient characteristics and postdosimetric parameters

	LDR-BT only (n = 276)	LDR-BT + EBRT (n = 202)	*p*
Age (year, median, IQR)	66.0 (62.0–70.0)	67.0 (63.0–71.0)	0.124
BMI (kg/m^2^, median, IQR)	23.4 (21.8–25.2)	23.7 (22.0–25.5)	0.348
PSA (ng/mL, median, IQR)	6.0 (4.9–7.7)	8.3 (5.7–11.7)	< 0.001
*Clinical T stage (number, %)*			
T1c	185 (67.0)	67 (33.2)	< 0.001
T2a	75 (27.2)	57 (28.2)	
T2b	7 (2.5)	23 (11.4)	
T2c	8 (2.9)	43 (21.3)	
T3a	0	12 (5.9)	
T3b	1 (0.4)	0	
*Gleason group grade (number, %)*			
1	175 (63.4)	20 (9.9)	< 0.001
2	95 (34.4)	76 (37.6)	
3	5 (1.8)	61 (30.2)	
4	1 (0.4)	28 (13.9)	
5	0	17 (8.4)	
*NCCN risk classification (number, %)*			
Low risk	154 (55.8)	0	< 0.001
Favorable-intermediate risk	85 (30.8)	41 (20.3)	
Unfavorable-intermediate risk	34 (12.3)	103 (51.0)	
High risk	3 (1.1)	51 (25.3)	
Very high risk	0	7 (3.4)	
Neoadjuvant ADT (number, %)	196 (71.0)	169 (83.7)	0.002
Prostate volume at LDR-BT (mL, median, IQR)	25.1 (19.2–31.0)	20.9 (16.8–27.2)	< 0.001
*Postdosimetric parameters*			
%D90 (%)	119.5 (111.4–126.3)	119.8 (111.2–128.2)	0.888
Prostate D90 (Gy)	173.1 (161.4–183.0)	124.6 (115.8–133.3)	< 0.001
BED (Gy)	183.6 (170.6–194.8)	208.2 (200.5–215.7)	< 0.001
% UD30 (Gy)	211.0 (194.3–2313)	154.1 (141.3–176.6)	< 0.001
Rectum V100 (cc)	0.3 (0.1–0.8)	0.3 (0–0.7)	0.429
Follow-up period (month, median, IQR)	103.0 (72.0–132.0)	110.0 (68.5–137.5)	0.652

*ADT* Androgen Deprivation Therapy, *BED* Biologically Effective Dose, *BMI* Body Mass Index, *EBRT* External Beam Radiation Therapy, *IQR* Interquartile range, *LDR-BT* Iodine-125 low-dose-rate brachytherapy, *NCCN* National Comprehensive Cancer Network, *PSA* Prostate specific antigen, *Prostate D90* Minimal dose (Gy) received by 90% of the prostate, *RectumV100* Rectal volume (cc) receiving 100% of the prescribed dose, *%D90* Minimal percentage of the dose received by 90% of the prostate gland, *%UD30* minimal percentage of the dose received by 30% of the urethra

### Proportion of patients with SPTs following LDR-BT

**Fig. 1:**
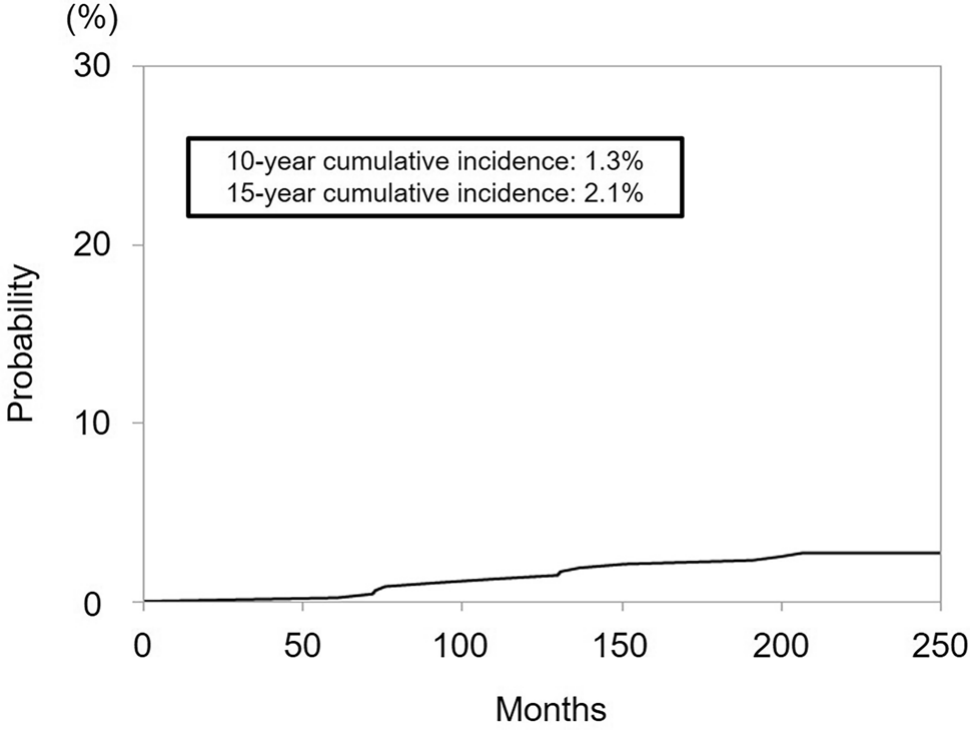
Cumulative incidence of secondary primary tumors (SPTs) following iodine-125 low-dose-rate brachytherapy. The 10 year and 15 year cumulative incidences of SPTs were 1.3% and 2.1%, respectively

**Fig. 2:**
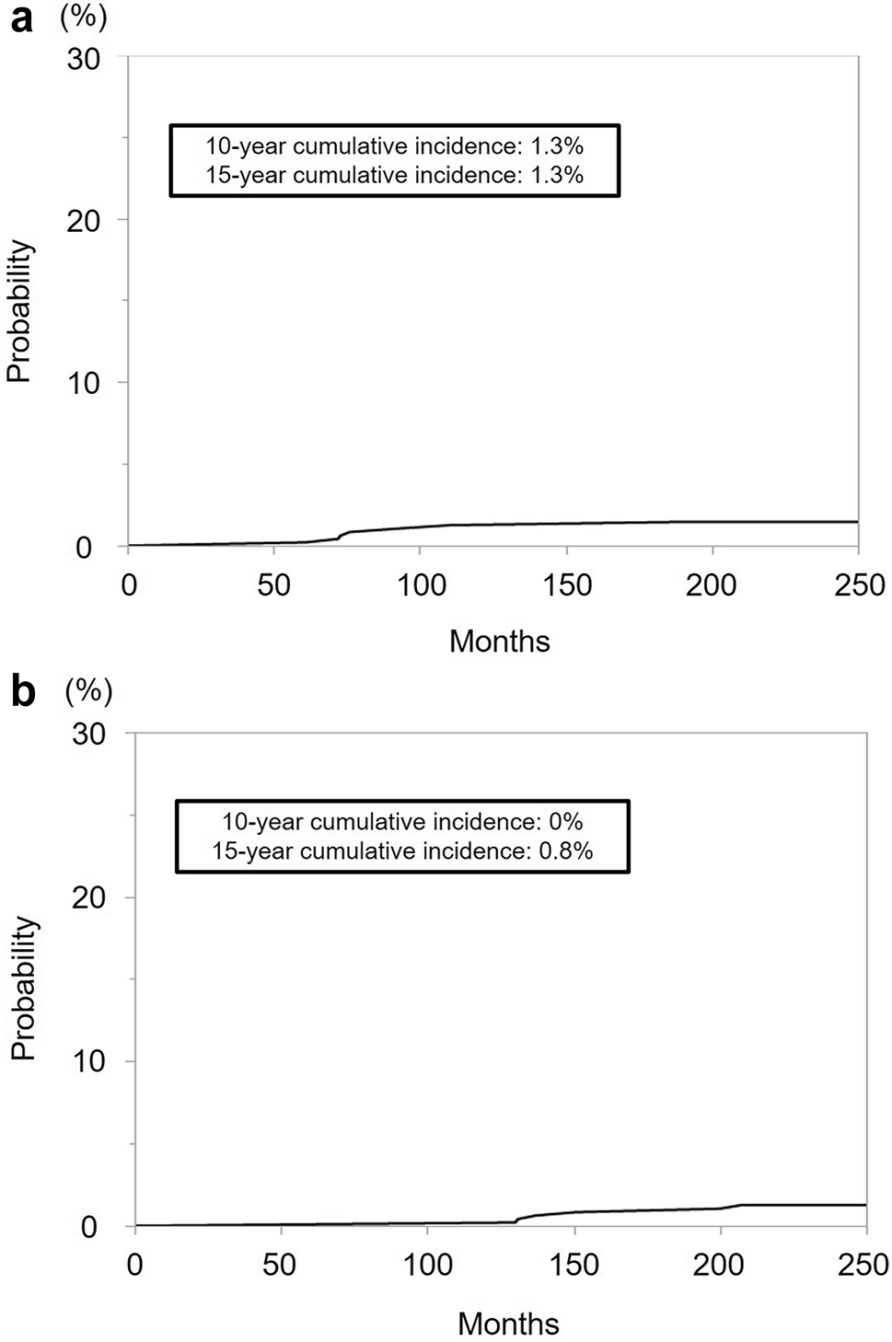
Cumulative incidence of bladder and rectal/anal canal cancers following iodine-125 low-dose-rate brachytherapy. **a** Cumulative incidence of bladder cancer. The 10 year and 15 year cumulative incidences of bladder cancer were 1.3% and 1.3%, respectively. **b** Cumulative incidence of rectal and anal canal cancers. The 10 year and 15 year cumulative incidences of rectal/anal canal cancers were 0% and 0.8%, respectively

Thirteen patients (2.7%) had SPTs. The median time to development of SPTs were 103.5 months (IQR: 71.0–132.0), and the 10 year incidence rate was 1.3% (Fig. 1). Seven cases of bladder cancer (1.5%) and six cases of rectal/anal canal cancer (1.3%) were documented. All bladder cancers were identified as urothelial carcinomas, with one case presenting glandular differentiation. Among rectal/anal canal cancers, four were classified as adenocarcinomas and two as squamous cell carcinomas. A specific histological profile was not observed among the patients in this cohort. The median time to development was 103.5 months (IQR: 71.0–132.3) for bladder cancer and 105.0 months (IQR: 71.0–133.3) for rectal/anal canal cancer. The 10 year incidence rates of bladder and rectal/anal canal cancers were 1.3% and 0%, respectively (Fig. 2). Of the patients studied, one died from bladder cancer and the other from rectal cancer. Among the seven patients who developed bladder cancer, three had a history of smoking, three were non-smokers, and smoking status was unknown in one patient.

### Predictive factors of SPTs following LDR-BT

**Fig. 3:**
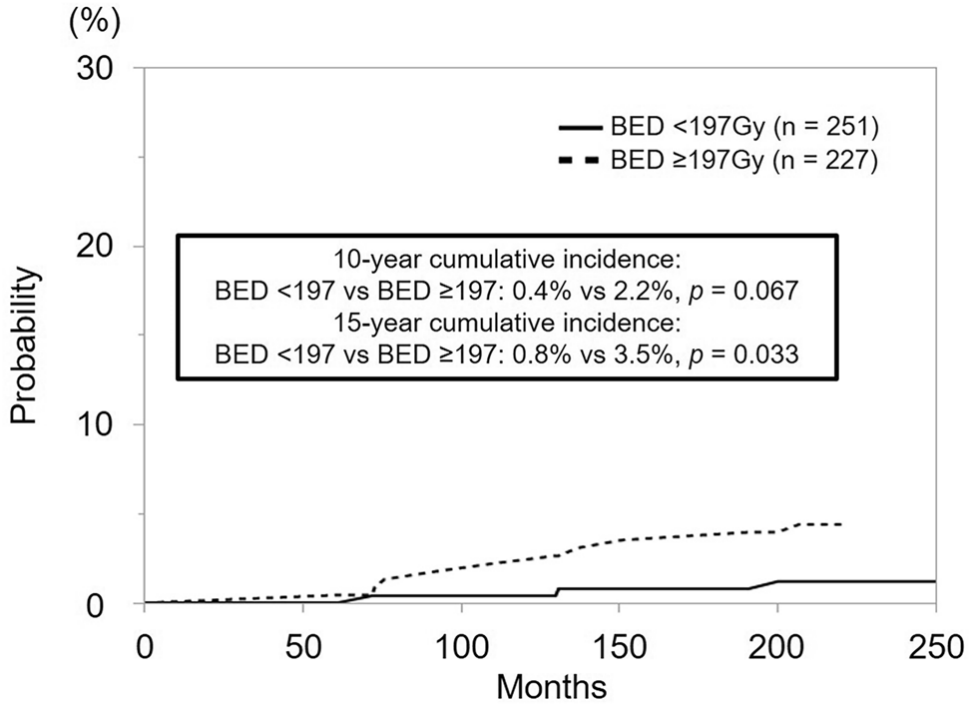
Cumulative incidence of secondary primary tumors following iodine-125 low-dose-rate brachytherapy stratified by biologically effective dose (BED). The 15 year cumulative incidence was significantly higher in patients with BED ≥ 197 Gy than in those with BED < 197 Gy (*p* = 0.033)

The 10 year cumulative incidence of SPTs tended to be higher in patients with BED ≥ 197 Gy than in those with BED < 197 Gy, and the 15 year cumulative incidence of SPTs were significantly higher in patients with BED ≥ 197 Gy than in those with BED < 197 Gy. (*p* = 0.033; Fig. 3). The 10 year and 15 year cumulative incidence rates of SPTs were 0.7% and 1.5% in the LDR-BT monotherapy group, and 1.5% and 3.0% in the combination LDR-BT and EBRT group (*p* = 0.654 and *p* = 0.335, respectively). Although a slightly higher tendency for SPTs incidence was observed in the combined LDR-BT and EBRT group, no significant difference was found. When patients were stratified by RV100 (< 0.64 mL vs. ≥ 0.64 mL), the 10 year cumulative incidence of SPTs were 0.9% in the < 0.64 mL group and 1.5% in the ≥ 0.64 mL group (*p* = 0.626). The 15 year cumulative incidence rates were 1.8% and 2.9%, respectively (*p* = 0.481). After thorough analysis, no substantial discrepancy in SPTs occurrence was noted between the two groups.

The associations between SPTs and clinical covariates are presented in Table 2. In an exploratory multivariable Cox regression model including age, BMI, NCCN risk category, neoadjuvant ADT, and BED, BED ≥ 197 Gy was associated with an increased risk of SPTs (hazard ratio [HR] 4.145; 95% confidence interval [CI] 1.108–15.498; *p* = 0.035; Table 2). In sensitivity analyses using a reduced multivariable model including only age and BED, BED ≥ 197 Gy remained significantly associated with SPTs (HR 3.842, 95% CI 1.050–14.062, *p* = 0.042). In the model, BED was represented as a continuous variable with adjustment for age. The analysis revealed that BED exhibited no statistically significant association with SPTs (HR per 10 Gy in-crease: 1.028, 95% CI 0.820–1.288).

**Table 2:** Predictive factors of secondary primary tumors in patients with localized prostate cancer who underwent Iodine-125 low-dose-rate brachytherapy: results of multivariate analysis

	Multivariate analysis			
	Number	HR	95%CI	*p*
*Age (year)*				
< 69	296	1 (ref.)	–	0.386
≥ 69	182	0.506	0.109–2.358	
*BMI (kg/m*^*2*^*)*				
< 26.0	397	1 (ref.)	–	0.465
≥ 26.0	81	0.465	0.059–3.628	
*NCCN risk classification*				
Low + Intermediate risk	417	1 (ref.)	–	0.995
Poor risk	61	0.993	0.120–8.239	
*Neoadjuvant ADT*				
No	113	1 (ref.)	–	0.138
Yes	365	0.399	0.119–1.342	
*BED (Gy)*				
< 197.0	251	1 (ref.)	-	0.035
≥ 197.0	227	4.145	1.108–15.498	

*ADT* Androgen Deprivation Therapy, *BED* Biologically Effective Dose, *BMI* Body Mass Index, *CI* Confidence interval, *HR* hazard ratio, *NCCN* National Comprehensive Cancer Network, *ref*. Reference

## Discussion

SPTs has been reported in numerous cases of malignant neoplasms treated with RT, including breast, oral, and cervical cancers, and lymphoma [24–26]. Radiation-induced DNA damage and long-term genomic instability are considered key mechanisms underlying SPTs development [27, 28]. The latency period for SPTs are generally considered to range from several years to decades, thereby underscoring the significance of prolonged follow-up in patients with cancer who have undergone RT therapy. Prolonged survival after definitive RT highlights the clinical importance of evaluating long-term SPTs risk.

Table 3 summarizes previous studies on LDR-BT with or without EBRT for PCa. The median follow-up period in these studies ranged from 69.6 to 165.6 months, with the reported incidence rates of SPTs ranging from 1.0 to 4.3% [29–34, 36]. A previous study examining the incidence of SPTs after LDR-BT in 897 Japanese patients found SPTs in 1.3% of the patients during a median follow-up period of 85.2 months [33]. Regarding the prevalence of various diseases, bladder cancer was observed in 1.0% of patients, while rectal cancer was identified in 0.3% [33]. A study examining the risk of bladder cancer development in 1,162 Japanese patients treated with LDR-BT alone or in combination with EBRT reported a bladder cancer incidence of 3.0% at a median follow-up period of 124.8 months [35]. In this study, the incidence of SPTs were 2.7% over a median follow-up period of 105.0 months. The incidence rates of bladder cancer and rectal/anal canal cancer were 1.5% and 1.3%, respectively. In our cohort, which has been followed for approximately 10 years, the incident rate of SPTs were consistent with that reported in previous studies that had comparable follow-up periods. However, the cumulative incidence of SPTs may increase significantly with longer follow-up periods. Previous studies with long-term follow-up have demonstrated a time-dependent increase in SPTs incidence [32, 36]. In these cohorts, the incidence of SPTs in the same patient population increased from 2.1% at a median follow-up of 69.6 months to approximately 7% after more than 14 years of follow-up. The present study indicates that the majority of SPTs cases occurred more than five years after LDR-BT, suggesting that longer-term, systematic follow-up is crucial. Specifically, the implementation of more frequent monitoring, including urine tests, fecal occult blood tests, and colonoscopies, between five and ten years after LDR-BT administration may lead to the early detection of SPTs.

**Table 3:** Previous reports on the development of secondary primary tumors in patients with localized prostate cancer who underwent Iodine-125 low-dose-rate brachytherapy

Author (Year)	Patients (Number)	Treatment (Number)	Follow-up period (Month, median)	Incident rate (Number, %)	Secondary cancer site (Number)
Liauw SL, et al. [29]	348	LDR-BT alone: 125 LDR-BT + EBRT: 223	126.0	15 (4.3)	Bladder: 1 Colorectal: 3 Prostatic urethra: 1
Musunuru H, et al. [30]	1805	LDR-BT alone: 1805	96.0	13 (1.0)	Bladder: 10 Rectal: 3
Hinnen KA, et al. [31]	1187	LDR-BT alone: 1187	85.2	26 (2.2)	Bladder: 9 Rectal: 17
Hamilton SN, et al. [32]	2418	LDR-BT alone: 2418	69.6	51 (2.1)	Bladder: 32 Rectal: 19
Nakai Y, et al. [33]	897	LDR-BT alone: 507 LDR-BT + EBRT: 390	85.2	12 (1.3)	Bladder: 9 Rectal: 3
Cosset JM, et al. [34]	675	LDR-BT alone: 675	132.0	14 (2.1)	Bladder: 9 Rectal: 5
Ozawa et al. [35]	1162	LDR-BT alone: 607 LDR-BT + EBRT: 555	124.8	–	Bladder: 27
St-Laurent MP, et al. [36]	2378	LDR-BT: 2378	165.6	15 years: 7.0%	Bladder: 107 Rectal: 51
Present study	478	LDR-BT alone: 276 LDR-BT + EBRT: 202	105.0	13 (2.8)	Bladder: 7 Rectal: 6

*EBRT* External Beam Radiation Therapy, *LDR-BT* Iodine-125 low-dose-rate brachytherapy

The relationship between LDR-BT and the risk of developing SPTs remains controversial. SPTs development after LDR-BT for PCa is associated with several risk factors, including advanced age, history of smoking, and the use of combination therapy involving EBRT [32, 37, 38]. Smoking is recognized as a well-established risk factor for developing bladder cancer [39, 40]. The present study indicates that both smokers and non-smokers developed bladder cancer subsequent to LDR-BT, thereby suggesting that radiation therapy may have influenced the subsequent carcinogenesis. However, smoking history was not documented for the entire cohort, which prevented its inclusion as a covariate in multivariate analysis and left unaccounted for potential bias. According to the findings of several single-center studies, SPTs incidence with LDR-BT is comparable to that of radical prostatectomy (RP) [31, 41]. Similarly, studies examining the risk of bladder cancer development found no significant differences between patients who received LDR-BT and non-RT for PCa [42, 43]. These reports suggest that LDR-BT alone may not increase the long-term risk of secondary primary malignancies. Conversely, St-Laurent et al. [36] reported an increased risk of second malignancies in patients undergoing LDR-BT compared with RP (HR 1.81, 95% CI 1.45–2.26, p < 0.001). Notably, their definition of second malignancies was not limited to radiation-associated tumors and included cases diagnosed within 5 years after treatment, which differs from the definition used in the present study. Furthermore, epidemiological data indicated an elevated prevalence of bladder and rectal cancer in patients diagnosed with PCa after LDR-BT [44]. In this study, higher BED (≥ 197 Gy) was associated with increased incidence of SPTs. Due to the small number of events, the results of this study should be regarded as exploratory. Radiotherapy as a definitive treatment has been shown to increase the likelihood of secondary malignancies irrespective of the primary tumor type [45, 46]. Inskip et al. [46] demonstrated a clear dose–response relationship between radiation exposure and the development of new primary solid cancers [45]. Specifically, elevated radiation doses have been demonstrated to markedly increase the likelihood of developing secondary malignant neoplasms irrespective of the primary cancer type [45]. Similarly, another study evaluated the correlation between therapeutic radiation and the likelihood of secondary malignancies in multiple organs [46]. This analysis revealed that radiation dosage plays a pivotal role in carcinogenesis irrespective of the specific tumor type [46]. However, the importance of increasing the BED to achieve favorable oncological outcomes after LDR-BT for localized PCa has been well-documented [47, 48]. The variability in the study results may be attributed to various factors, including treatment-related conditions, follow-up periods, patient backgrounds, dosimetric parameters, and monitoring intensity. Patients who have undergone high-dose LDR-BT require meticulous and prolonged follow-up to ensure optimal outcomes and mitigate potential complications. The achievement of a BED exceeding 197 Gy with LDR-BT monotherapy alone is a rare achievement, and the administration of additional EBRT is common in most cases. However, no statistically significant difference was observed between the LDR-BT monotherapy group and the LDR-BT + EBRT combination group at either the 10 year or 15 year follow-up points. These findings suggest that while adding EBRT may increase the radiation dose to pelvic tissues, the risk of developing SPTs are not solely attributable to the additional EBRT irradiation. Conversely, the development of bladder or anorectal cancer is anticipated to occur at a certain rate in elderly men, regardless of their history with radiation therapy. Therefore, a critical aspect of interpreting these findings is recognizing that the SPTs observed in this cohort cannot be attributed exclusively to radiation exposure.

This study has several limitations. First, it was a retrospective, single-center analysis, which may limit generalizability. Second, the number of SPTs events were small, and multivariable analyses should therefore be interpreted as exploratory. Third, smoking history, an important risk factor for bladder cancer, was not systematically available for the entire cohort and could not be included in multivariable analyses. Fourth, detailed dose–volume analyses of organs at risk were not performed and may provide additional insights in future studies. Fifth, this study exclusively included patients with PCa who underwent LDR-BT and did not compare them with those receiving other treatment modalities, including surgery or hormone therapy. Consequently, a large-scale, multicenter, prospective study is necessary for an accurate assessment of the incidence rate of SPTs after LDR-BT. Finally, the absence of a non-irradiated control group or age-matched general population data precluded direct estimation of excess radiation-attributable risk. Moreover, this study did not examine the effects on SPTs at relatively low doses, such as RV30 and RV50. Nevertheless, given the potential association with SPTs in LDR-BT combined with EBRT, further investigation may be warranted.

## Conclusions

In this long-term retrospective analysis of Japanese patients with localized PCa treated with LDR-BT with or without EBRT, SPTs incidence was found to be low. Although an increase in the incidence rate of SPTs occurrence was observed at BED ≥ 197 Gy, this finding might require careful interpretation due to the limited number of events. These findings emphasize the critical need for meticulous and sustained post-treatment monitoring, particularly in patients undergoing high-dose LDR-BT. Early detection and timely intervention for SPTs may help maintain favorable long-term oncological outcomes and minimize the potential impact of SPTs on patient prognosis.
